# Torpor: The Rise and Fall of 3-Monoiodothyronamine from Brain to Gut—From Gut to Brain?

**DOI:** 10.3389/fendo.2017.00118

**Published:** 2017-05-31

**Authors:** Hartmut H. Glossmann, Oliver M. D. Lutz

**Affiliations:** ^1^Institut für Biochemische Pharmakologie, Innsbruck, Austria; ^2^Austrian Drug Screening Institute, Innsbruck, Austria

**Keywords:** T1AM, thyroxine, monoiodothyronamine, apolipoprotein B-100, hibernation, immunoassay, torpor, mass spectrometry

## Abstract

**3-Monoiodothyronamine** (T1AM), first isolated from rat brain, is reported to be an endogenous, rapidly acting metabolite of thyroxine. One of its numerous effects is the induction of a “torpor-like” state in experimental animals. A critical analysis of T1AM, to serve as an endogenous cryogen, is given. The proposed biosynthetic pathway for formation of T1AM, which includes deiodinases and ornithine decarboxylase in the upper intestinum, is an unusual one. To reach the brain *via* systemic circulation, enterohepatic recycling and passage through the liver may occur. The possible role of gut microbiota is discussed. T1AM concentrations in human serum, measured by a specific monoclonal assay are up to three orders of magnitude higher compared to values obtained by MS/MS technology. The difference is explained by the presence of a high-affinity binder for T1AM (Apolipoprotein B-100) in serum, which permits the immunoassay to measure the total concentration of the analyte but limits MS/MS technology to detect only the unbound (free) analyte, a view, which is contested here.

## 1 Introduction

Hibernation has fascinated scientists for centuries (1–3). Obligate hibernators, e.g., the 13-lined ground squirrel (*Ictidomys tridecemlineatus*) can survive many months under harsh conditions (no food and very low ambient temperatures) in winter. They recover in spring, apparently without any functional or organ damage. The squirrel enters this state, termed torpor, by rapidly decreasing its metabolism and lowering the core temperature down to 3 or 4°C. After a few weeks in torpor, brief periods (12–24 h) of “interbout euthermia”, which are essential for survival, are observed (4). The processes of hibernation (entry and arousal) are of interest, e.g., for organ preservation in transplantation (5), ischemia–reperfusion damage and cardio protection in the context of cardiac surgery (6, 7), organ protection after hemorrhagic shock or global ischemia after cardiac arrest (8), and protection of brain from ischemic injury (9). Likewise, understanding the conservation of bone and skeletal muscle mass and performance (10) despite many months of inactivity may be of interest for NASA, planning a Mars flight, perhaps with a “torpid” crew (11). A torpor-like state, but not multiday hibernation, can be induced in mice by fasting and a cage temperature below their thermoneutral zone. The search for endogenous signals that trigger hibernation or a torpor-like state started in 1969 (12) and remains topical, with the aim of developing drugs for therapeutic hypothermia. Among the endogenous compounds mentioned in reviews (13, 14) is 3-monoiodothyronamine (T1AM). This paper shall serve as a focused review on torpor induction by T1AM in the context of its pharmacology and the mysteries of its biological origin.

## 2 Exciting Properties of the Novel, Thyroxine-Derived, Hormone 3-Monoiodothyronamine

“*These unique molecules* [i.e. T1AM] *have developmental potential as cryogens for the treatment of stroke, in which rapid and prolonged cooling offers outstanding therapeutic benefit to patients*” (15). “*Such potent actions of 3-T1AM, its metabolites, and synthetic congeners are of eminent interest in emergency and critical care medicine, surgery, tissue transplantation, metabolic and eye clinics, as well as space science. Application of an endogenous biogenic cryogen derived from a hormone provides a rather safe and valuable “lead compound” to be tested and developed by the pharmaceutical industry for various medical applications*…” (16). “*From the current body of literature, potential therapeutic applications with T1AM are quite apparent, ranging from sleep/torpidity induction, conferring protection against ischemic injury, and anti-obesogenic by inducing increased metabolic reliance on lipid oxidation*” (17). “*The major endogenous thyroid hormone metabolite 3-iodothyronamine (3-T1AM) exerts marked cryogenic, metabolic, cardiac and central nervous system actions. It is bound to apolipoprotein B-100 (ApoB-100), possibly facilitating its cellular uptake via interaction with the low density lipoprotein-receptor*” (18).

## 3 Trace Amine-Associated Receptors and 3-Monoiodothyronamine

T1AM, after its isolation from rat brain, was tested as a putative endogenous ligand (19) for activation of trace amine-associated receptors (TAARs) (20, 21). Trace amines (e.g., octopamine, tyramine, *β*-phenylethylamine) received their name from their two to three orders of magnitude lower abundance in brain tissue compared to classical amine neurotransmitters such as noradrenaline, serotonin, or dopamine.

Trace amines are formed enzymatically from aromatic amino acids by decarboxylation and were, for a long time, regarded as curiosities. The situation changed when high-affinity binding sites were identified in brain membranes by classical grinding and binding experiments with radiolabeled trace amines. The sensitivity of these agonist binding sites to guanylyl nucleotide inhibition indicated their relationship to the family of G*αs* G protein-coupled receptors, which finally led to cloning of the first prototype TAAR1 of a larger family in 2001. For a review of its discovery and properties, we recommend the review by Grandy (22). Except TAAR1, all other TAARs function as odorant receptors expressed on olfactory neurons. Mice possess 14 of such receptors in their nose epithelium (23) compared to five TAARs in humans. Among them is human TAAR5, which is activated by trimethylamine, occurring in rotten fish (24) and TAAR2, which also occurs in human white blood cells (25) and in mucosal layers of the gastrointestinal tract of mice (26). TAAR1 protein is expressed in brain but also in the periphery [e.g., heart, T-lymphocytes, stomach (27), duodenum, and pancreatic *β*-cells (28, 29)]. Human TAAR1 is implicated in drug addiction, eating behavior, sleep-wake balance, and neuropsychiatric disorders (30, 31). This explains the initial excitement for T1AM that was postulated to be a metabolite of T_4_ and an endogenous physiological signal acting rapidly *via* cell surface receptors similar to the actions of T_4_ and T_3_ on *α_V_β*_3_ integrins (32).

Injecting T1AM into mice induces a “torpor-like” state. This immediately fascinated scientists and even convinced a National Space Lab program in South Korea to fund research on the newly found “hibernating” drug (33).

## 4 Pharmacodynamics of T1AM

Rodents such as mice and rats have a large surface area compared to their volume, consequently suffering from much greater heat loss compared to larger animals (34). At cage temperatures below thermoneutrality (about 28°C for rats and 30°C for mice), the sympathetic nervous system and brown adipose tissue (BAT) are always activated (35), and a considerable fraction of the total energy expenditure is spent for cold-induced thermogenesis *via* BAT. Many small mammals have a natural defense mechanism during the colder season, upon a decline in food supply (36). The set point is lowered in the hypothalamus, and the core temperature approaches ambient temperatures. This “torpor-like” state may not be confused with hibernation (4) but is nevertheless often used as a readout for drug candidates investigated for therapeutic hypothermia. Among them are adenosine agonists (37–39) and *α*_2_ adrenergic agonists (40).

Upon intraperitoneal injection of T1AM, the rectal temperature of mice dropped in a dose-dependent manner with an ED50 of around 25 mg/kg. Mice injected with 100 or 200 mg/kg died, suggesting a very small or even absent therapeutic window for torpor induction (19). Moreover, the heart rate dropped significantly, and a strong negative inotropic effect was observed in the isolated perfused rat heart preparation. The T1AM-treated mice are sedated, have a hunched back, closed eyes, and the tail rolled around the body. This “hibernated” state could be reproduced several times by multidose application, provided the animals were always warmed up between the applications (33). Despite their sleep-like state, T1AM-receiving rodents are under extreme metabolic stress: plasma levels of corticosterone, glucagon, and glucose increase several-fold in rats, but insulin is not responding to the raised glucose levels (41). The combination of sedation, bradycardia, and hypothermia in rodents is typical for centrally acting *α*_2_ adrenoceptor agonists such as the approved drugs clonidine, guanabenz, the sedo-analgesic dexmedetomidine, or the Servier experimental compound S18116, which is one of the most potent and selective drugs from this class (42).

These agonists decrease sympathetic outflow from the brainstem and inhibit BAT thermogenesis in rodents (40). Guanabenz was able to maintain the torpid state in rats for up to 7 days without warming-up periods (43). Subcutaneous S18116 injection lowers the body temperature of mice by up to 10°C in the dose range from 0.1 to 40 µg/kg, with an EC50 value of 2.5 µg/kg (42). None of the aforementioned drugs killed the animals at acutely effective hypothermic doses, indicating a sufficiently high therapeutic index for this effect. Unfortunately, quantal dose–effect curves for T1AM are not available but exist for all of the drugs mentioned. T1AM is a highly potent agonist for the receptor subtype *α*_2_*A* (29). This can explain the inhibition of insulin release despite hyperglycemia (41) and provides the most likely mechanism by which the rapid drop in core temperature occurs. Blockade of the sympathetic outflow from the brain by T1AM inhibits heat generation by BAT, which is further enhanced by low cardiac output. All of the mentioned *α*_2_ adrenoceptor agonists had much wider hypothermic windows than T1AM. The large number of metabolites (see Section 6) and already demonstrated additional targets possibly contribute to the pronounced toxicity. An excellent study with NMR-based metabolomics in obese mice (44) supported earlier findings in rats (41). Upon chronic application of T1AM (10 mg/kg/day) for 7 days, the compound initially increased lipolysis and *β*-hydroxybutyrate concentrations in plasma, indicating acute inhibition of insulin secretion. On days 5–7, a shift from lipid oxidation to either carbohydrate or protein metabolism as macronutrients was observed. After 7 days, the T1AM-treated mice, in contrast to the vehicle-treated animals, were still not gaining weight for additional 14 days and had increased valine and glycine concentrations in plasma. The authors commented on these toxic posttreatment effects as follows: “*The discovery that protein catabolism induction can occur after chronic application of T1AM at low concentration is important and demonstrates the power of combined analyses for anti-obesity drug evaluations to identify unexpected side effects*” (44).

Contrary to earlier expectations, TAAR1 is not responsible for the T1AM-induced torpor-like state. TAAR1 knockout mice still respond to the compound by lowering their body temperature. Classical activators of these receptors even increase the core temperature (45). Peripheral signals for the adaptive behavior of rodents upon fasting in a cold environment have been identified: lower leptin and insulin, higher ghrelin and uridine (46, 47) signal to the hypothalamus for a decrease of the temperature set point. Compared to *α*_2_*A* receptor agonists (either approved or experimental), adenosine, its analogs or uridine (48), the low potency of T1AM to induce a torpor-like state in comparison to its toxic effects disqualify it as an “*endogenous biogenic cryogen*” (16).

## 5 *In Vitro* Pharmacodynamics of T1AM

Synthetic T1AM is a potent activator of rat (EC50: 14 nM) and mouse (EC50: 112 nM) TAAR1, stably expressed in HEK-293 cells (19). In another cell line, the EC50 was determined as 22.4 nM for the rat and 1,510 nM for human TAAR1. These values may be compared to those of *β*-phenylethylamine, which activates human TAAR1 with an EC50 of 106 nM and rat TAAR1 with 206 nM (49). The low potency of T1AM for human TAAR1 was confirmed by others (EC50: 1,690 nM) (50). It is therefore unlikely that there exist any significant effects in humans *via* TAAR1. A useful comparison of these values may be drawn with data for a selective ligand, RO5166017 [(S)-4-[(ethyl-phenyl-amino)-methyl]-4,5-dihydro-oxazol-2-ylamine]. RO5166017 has EC50 values in HEK-293 cells of 3.3 nM (mouse), 2.7 nM (rat), and 31 nM (human) (51). In addition to a favorable pharmacokinetic profile, a radioligand screening of RO5166017 against 123 target proteins revealed little or no interaction with other receptors, transporters, or enzymes. To prove that the effects of such selective drugs on, e.g., animal behavior (51) or metabolism (28) are indeed mediated by TAAR1 and not *via* “off-targets”, the TAAR1 knockout mouse is employed as a negative control. Alternatively, actions could be blocked by a selective TAAR1 antagonist (52).

With respect to the target profile of T1AM, *α*_2_ adrenoceptors are activated with a similar potency as noradrenaline. Neuronal membrane as well as vesicular transporters for dopamine and noradrenaline (45, 53) and all subtypes of muscarinic acetylcholine receptors (54) are functionally blocked in the micromolar or sub-micromolar range. Other identified “targets” are cited in review articles (16, 17, 55). One very high-affinity binding site, apolipoprotein B-100 (ApoB-100), is mentioned here for two reasons. First, ApoB-100 is suggested to be relevant for delivery of the novel hormone to tissues (18) and second, its seemingly problematic nature in the context of accurate quantification of T1AM *via* MS/MS technology (56) deserves mention. ApoB-100 is a component of circulating VLDL and LDL lipoproteins and binds to T1AM in a 1:1 stoichiometry with a K*_d_* of 17 nM (57). The concentration of serum ApoB-100 in healthy adults ranges from 500 to 1,500 nM but is considerably lowered in patients undergoing statin therapy.

In sum, T1AM may be genuinely termed as a “multi-target” compound, or in plain words: it is a “dirty” drug.

## 6 *In Vivo* Pharmacokinetics

Unfortunately, the pharmacological profile is worsened upon a review of T1AM’s pharmacokinetics and metabolism in rodents. A great deal of effort was spent with MS/MS technology to elucidate the fate of injected T1AM in rodents (58). After intraperitoneal injection, the parent drug is rapidly cleared from plasma with an apparent half-life of 7–8 min during the first hour. Thereafter, a slower elimination with a half-life of about 50 min takes place (59). Oxidative deamidation by monoamine oxidase to 3-iodothyroacetic acid (TA1) (60, 61), glucuronidation, sulfation (62), acetylation, and deiodination are observed. Within 3 h after a single intraperitoneal injection, the sum of the three main metabolites in the serum of mice is approximately 3 µM. At this time, the concentration of the parent compound (T1AM) is reduced by approximately two orders of magnitude from 16.6 to 0.19 µM. Moreover, the concentration of TA1 (17.7 µM) is equal to that of T1AM (16.6 µM) after 10 min. The authors were surprised about the extent and speed of T1AM breakdown: “[A] *rich, diverse metabolism such as this is not generally seen with synthetic drugs or xenobiotics*” (58).

In conclusion, suggestions regarding the value of such properties as a lead compound for further cryogenic drug development will probably not convince the pharmaceutical industry.

## 7 T1AM is Claimed to be an “Endogenous” Derivative of Thyroid Hormone—But Where and How is it Made?

### 7.1 Tissue Distribution of T1AM

The claim of T1AM’s endogenous nature was supported by its presence in extracts from rat brain and liver, heart as well as blood samples from mice. Mass-spectrometric fragmentation of the isolated biological material and the synthetic compound yielded identical fragmentation patterns. No absolute concentrations were presented in 2004 (19), but the concentration of T1AM in drug naïve rat serum was later reported as 300 pM, in rat liver as 93 pmol/g, in rat brain cortex with 60 pmol/g, in rat kidney with 36 pmol/g (63), and in mouse liver with 2.4 pmol/g (64). Others reported 5.4 pmol/g in rat liver (65) or less than 0.3 pmol/g (66). In mouse brain, 48.3 pmol/g has been reported (60, 67) but in mice lacking histidine decarboxylase, T1AM could not be identified. In the corresponding wild type, 0.22 pmol/g were found. In the Djungarian hamster (*Phodopus sungorus*), the serum concentration was determined as 6 nM, increasing 3 h after intraperitoneal injection of 50 mg/kg T1AM about 10-fold (68). Presence of T1AM in brain homogenates of the hamsters was mentioned, but not reported in absolute concentrations.

Taken together, such high variability in the range of orders of magnitude is quite unusual for a T_4_ derived metabolite. T_4_ and T_3_ levels are fairly constant in human plasma with very small circadian variation (69). Analytical errors can be excluded since, for example, the mice brain concentrations, ranging from 0 to 48.3 pmol/g, have been reported by the same laboratory for three different mouse strains. The fact that rat liver concentrations are 300-fold higher than serum concentrations, again obtained by the same laboratory, argue toward the liver as receiving input not primarily from the hepatic artery but from the portal vein. With respect to other mammals, the presence of T1AM in brains of guinea pigs was mentioned but no absolute amounts were reported (19). Human thyroid tissue does not contain a trace of T1AM (i.e., <0.30 pmol/g) (66). As other tissues, including those from ruminants, are easily available, a lack of such investigations is surprising, possibly pointing to special features of rodents that are often not considered of relevance for metabolite research.

### 7.2 Mass Spectrometry

Multiple reaction monitoring mass spectrometry (MRM-MS) or higher stage fragmentation techniques such as MS^3^ of selected precursor ions serve as the golden standard for quantification of endogenously formed or exogenously acquired compounds occurring in trace amounts in biological matrices such as plasma, serum, or tissues. In the context of quantification, synthetic T1AM and a deuterated analog, serving as an internal standard, are available. They facilitate the calculation of recovery during extraction and aid the determination of the ionization efficiency during mass-spectrometric analysis. Obviously, they also enable the exact determination of the analyte’s retention time during chromatographic separation, preceding the mass-spectrometric analysis. The lower limit of detection (LOD) for T1AM in serum or plasma was reported as 250 pM (66) or “…*lower than 300 pM*” (63). Later, an LOD of 35 pM (70) was reported, with human patients exhibiting an average T1AM concentration of 219 pM, ranging from 160 to 300 pM. Ackermans et al. could not discover any T1AM above their LOD in serum or plasma of eight human volunteers (66). The possible lack in recovery of ApoB-100-bound T1AM was properly accounted for by employing an extraction protocol including protein digestion *via* Proteinase K. Even more disturbing is the fact that Ackermans et al. could easily detect T1AM in the serum or liver of T1AM-treated animals but never in serum or liver of rats treated with vehicle (66). Their LOD in tissues was reported at 0.30 pmol/g, one to two orders of magnitude below the amounts found in rat and mouse livers by Scanlan’s group. The latter laboratory commented the negative result as follows: “*Of note, a study designed similarly to ours was recently attempted and failed, because the investigators were unable to extract and detect endogenous T1AM by LC-MS/MS (32)*” (64). The above comment could be justified if the Scanlan laboratory supplied their tissue samples to the Amsterdam Laboratory of Endocrinology, which apparently never happened. As ApoB-100 is a major binder of T1AM, which is claimed to prevent sufficient extraction for the subsequent MS/MS analysis, it is noted that the rat liver, despite much lower production when compared to human liver, contains 146 mg/g of protein of ApoB-100 (71). After conversion into grams of wet weight, this amounts to about 60 nmol/g, which is three orders of magnitude higher compared to the highest levels ever reported for T1AM, being sufficient to bind an equal amount of T1AM. Moreover, Ackermans et al. ensured quantitative recovery of protein-bound T1AM in tissue samples by denaturing the proteins with acetic acetone. For the reasons given, the presence of ApoB-100 in the matrix cannot be made responsible for not discovering the analyte. For a detailed review addressing the major pitfalls in the quantification of thyroid hormone metabolites including T1AM, the reader is referred to the work of Richards et al. (72).

The published tissue and serum concentrations (see Section 7.6 for stability of T1AM in humans and (59) for rodents) are most likely correct. A few hypotheses are offered as explanations for the failure of Ackermans et al. to discover T1AM in the liver of vehicle-treated rats: firstly, Ackermans et al. kept the animals under different conditions as the Scanlan group, probably restrained and treated with antibiotics (73). Secondly, food or drinking water possibly contained traces of compounds which interfered with intestinal enzyme activities (e.g., deiodinases and ornithine decarboxylases), now shown to be involved in the biosynthesis of T1AM. Finally, levels in the liver are always a snapshot. If the input by whatever source occurred hours before the animals were sacrificed, the rapid degradation of T1AM lowered the amount below the reported detection limit.

### 7.3 T1AM Is Not Derived from Circulating Thyroxine

Two independent laboratories agree on the following, namely, that no peripheral or CNS conversion of injected T_4_ into T1AM occurs in rodents. One group delivered T_4_ as a ^13^C-labeled compound (^13^C_6_-T_4_) for 10 days with increasing doses by an osmotic minipump to rats, inducing different degrees of hyperthyroidism. They also employed the respective ^13^C-labeled triiodothyronine (T_3_) as a standard (66), but not a trace of newly formed ^13^C-labeled T1AM was observed in serum or the CNS. Interestingly, this important result is almost never given credit to and was also not mentioned by Hackenmueller et al. (64). Hackenmueller et al. used^13^C_9_-^15^N-T_4_ (“heavy T_4_”) and the respective standards after induction of hypothyroidism in mice by feeding perchlorate and methimazole. The various explanations of their negative result are worth being read in the original publications but will not be discussed here, especially in view of the interpretation given later by Hoefig et al. (74). In conclusion, there is no doubt that T1AM in rodents does not originate from circulating T_4_ under conditions of drug-induced hypothyroidism or various stages of T_4_-induced hyperthyroidism.

### 7.4 Ornithine Decarboxylase—The Missing Link

The entire enzymatic activity necessary to produce T1AM from T_4_ was shown to exist in intestinal tissue of mice (74). The tissue contained the enzyme ornithine decarboxylase (ODC) that is capable of decarboxylating T_4_ and its deiodinated intermediates. For analysis, the *ex vivo* everted gut sac model (jejunum) from pathogen free, but not axenic, mice was used. When the preparation (luminal side out) was incubated in solutions containing T_4_, significant amounts of T1AM were produced that could be identified *via* mass spectrometry. *In vitro* human ODC was able to decarboxylate 3,5-T_2_ to 3,5-T2AM and a possible sequence of enzymatic reactions leading to T1AM was presented. The authors attempted to explain the negative results by Hackenmueller et al. (64) and observed that a combined treatment with perchlorate and methimazole inhibited the intestinal expression of deiodinase 1 (DIO1) and ODC genes, which was not reversed by T_4_. However, an explanation for the negative results regarding the hyperthyroid rats (66) was not offered.

### 7.5 Gut Microbiota, Cecotrophy, Coprophagy

In a recent review article (16), it is proposed that for T1AM formation, T_4_ must enter the gut and may not be formed enzymatically elsewhere. This could indeed explain the high levels observed by some researchers in the rat liver. But this may not be the end of the story, especially considering additional important players: the gut microbiota (75). It is long known that deiodination and degradation of T_4_ and T_3_ by gut microbiota occur in the intestine of rodents (76). Moreover, when one partially decontaminates rats by feeding ampicillin, the metabolism of T_4_ and T_3_ is drastically changed (77). The intestinal wall contains ODC, but rodent gut microbes in the cecum and colon are another excellent source as *Klebsiella, Pseudomonas*, and *E. coli* (78) feature abundant constitutive and inducible (i.e., biodegradative) ODC. These enzymes appear to differ in some respect from the mammalian counterparts as the potent irreversible blocker difluoromethylornithine, currently in clinical cancer trials (79), inhibits ODCs of *Pseudomonas aeruginosa* but not those of *Klebsiella pneumoniae* and *E. coli* (80). Furthermore, there are enzymes in the gut microbiota of humans, and perhaps rodents, that are capable of decarboxylating aromatic amines (81). It is suggested to analyze T1AM in germ-free rats or axenic mice to refute the hypothesis about the role of gut bacteria.

If, as speculated here, the gut microbiome plays a significant role, a specific behavior of mice and rats may have contributed, namely the consumption of soft (or night) feces, originating from microbiota-digested cecum content. Rats ingest between 50 and 65% of their feces (82), which can enable several passages of T4 metabolites. The use of anti-coprophagy cages was not specifically mentioned in the publications about T1AM.

### 7.6 Serum Levels in Humans

With respect to humans, a chemiluminescent immunoassay (LIA) with a mouse monoclonal antibody for T1AM was developed. A median concentration of 66 nM in sera from healthy individuals and of 120 nM in thyroid cancer patients substituted with oral thyroxine was reported (56). In some of these patients, excessive amounts of up to 240 nM have been quantified. Most surprising was that for 10 T_4_-substituted patients with pituitary insufficiency, when tested 6 days after withdrawal, the initially observed T1AM levels did not change (i.e., 97 compared to 92 nM). Somewhat lower concentrations (14.5 nM) were observed in patients undergoing heart surgery (83). For this LIA, the capture antibody for the mouse monoclonal antibody originated from goat and the reporting label was horseradish peroxidase, coupled to T1AM. Unfortunately, problems with this assay do exist, as heterophilic antibodies, especially human anti-mouse antibodies (HAMAs), may interfere (84). For reasons unknown, many cancer patients feature HAMAs (85). But a more serious problem is the human serum itself, introducing the very high-affinity binder, ApoB-100 in significant concentrations. It is suggested here that HAMAs, ApoB-100 as well as related lipoproteins observed in LDL with a K*_d_* of 48 nM (57) may be responsible for these data, which differ from the MS/MS results by three orders of magnitude.

More recently, healthy controls were shown to have a median concentration of 8 nM whereas intensive care patients, often treated with antibiotics, had 4.8 nM (86). Here, the assay conditions have been changed as follows: the surface-bound capture is now T1AM, coupled to albumin, the mouse antibody is biotinylated, and the discovery system is Streptavidin-Europium. The authors mention ApoB-100 concentrations, apparently well aware of the aforementioned problems with the original assay. MS/MS measurements reported that T1AM concentrations in human sera or plasma are far below 1 nM (70). Roy et al. proved excellent stability of deuterated and non-deuterated T1AM in pooled human serum by incubating it for 24 hours at 37°C (57). However, in fetal bovine serum, which is often employed for cell culture experiments, pre-analytical degradation, different for internal standard and analyte (isotope effect), is suggested by preliminary experiments (87). The difference to rodent and human sera may be explained by the very high activity of soluble amine oxidases in bovine plasma. Their activity is very low in healthy humans and almost absent in rodent plasma (88).

It is hence anticipated that the ambiguities in the literature are resolved in the near future, especially in the context of proper sample preparation techniques and in adherence of analytical guidelines such as those published by the FDA or the EMEA. The publication by Rathmann et al. (89) serves as an excellent example of a thoroughly validated analytical method in this context.

## 8 Conclusion

**Figure 1:**
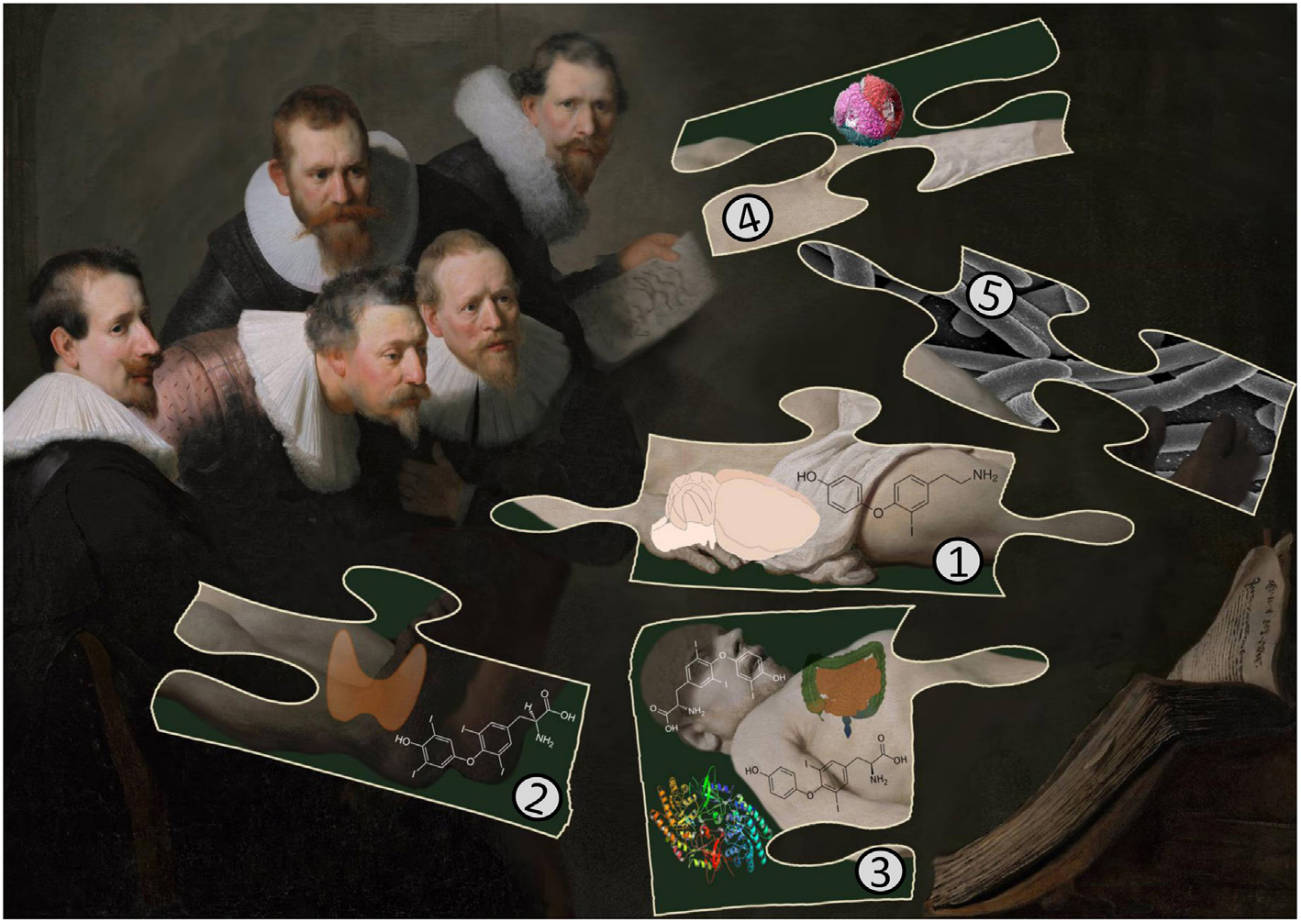
The cartoon illustrates important findings in T1AM research as a puzzle: (1) T1AM was isolated first from rat brain and later shown to be present in liver and other rodent tissues. Surprisingly, there are no quantitative data available reporting T1AM contents in other mammalian tissues. (2) The thyroid secretes T_4_ and T_3_ but human thyroid tissue does not contain T1AM. (3) Rodents cannot convert externally administered T_4_, labeled with ^13^C or 13C and ^15^N, into T1AM but T_4_ is converted by mouse jejunal tissue into T1AM. The enzyme responsible for decarboxylation from deiodinated T_4_ intermediates is ornithine decarboxylase (ODC). (4) Apolipoprotein B-100 in LDL is speculated to transport T1AM to target tissues and suggested to interfere with the correct determination of T1AM human serum concentrations with MS/MS but not with immunoassays [*Image adapted from Kumar et al. (98)*]. (5) The role of the gut microbiota in the biosynthesis of T1AM is unclear but speculated to be of importance.

The discovery of T1AM as an endogenous novel metabolite of T_4_ exemplifies the great analytical power of MS/MS technology to identify and quantify molecules occurring in various matrices, including human plasma or animal tissues at incredibly low concentrations. The exciting finding that T1AM activates rodent TAARs with nanomolar EC50 values (19) stimulated further research. A breakthrough in the mysterious biological pathway regarding the formation of T1AM was that in addition to deiodinases such as DIO1, ODC is involved (74). One may conclude that the rise of fame of T1AM started with a rat brain extract and ended in the gut, but the question of a route from gut to brain remains (see Figure 1). T1AM shares an analogy with another hormone, abscisic acid (ABA). This phytohormone was isolated from pig and rat brain in 1986, guided by a highly specific antibody. The identity of ABA was proven by MS/MS and the purified compound was shown to be functionally active in a conventional ABA bioassay (90). The authors were surprised about the presence of a phytohormone in mammalian brain and kept rats on an ABA-deficient diet for a long time. To their surprise, the ABA diet-deficient rats almost doubled the content in the brain, suggesting that ABA is possibly synthesized in the absence of external supplies. Some years later, others discovered greatly reduced ABA concentrations in brain samples of ruminants but confirmed the high concentrations in rodents (91). As an explanation, the authors pointed out that ruminants had bacteria in the upper intestine whereas rats have them in the distal part. Indeed, ABA is produced by gut bacteria (92, 93). Before discarding these citations as completely irrelevant to T1AM, one should take notice that ABA is circulating in human plasma in nanomolar concentrations. ABA is not an inert contaminant from plant-derived sources. Instead, it is a powerful regulator of glucose metabolism in humans in doses of a single microgram per kilogram (94–97). One can agree with Hoefig et al. (16), that it will take much less time today compared to earlier discoveries, to unravel the mysteries of the novel T_4_ metabolite.

## Author Contributions

HG collected the data; HG and OL wrote the paper; OL provided assistance during production of the final format of the manuscript for readers not acquainted with the subject.

## Conflict of Interest Statement

The authors declare that the research was conducted in the absence of any commercial or financial relationships that could be construed as a potential conflict of interest.
