# Erratum: Torpor: The Rise and Fall of 3-Monoiodothyronamine from Brain to Gut—From Gut to Brain?

**DOI:** 10.3389/fendo.2017.00158

**Published:** 2017-07-10

**Authors:** Frontiers Production Office

**Affiliations:** ^1^Frontiers Media SA, Lausanne, Switzerland

**Keywords:** T1AM, thyroxine, monoiodothyronamine, apolipoprotein B-100, hibernation, immunoassay, torpor, mass spectrometry

**Reason for Erratum:**

Due to typesetting errors, three mistakes were introduced in the published article.

In the section “7.5. Gut Microbiota, Cecotrophy, Coprophagy”, page 6, left column, 2nd line, should read:

Correct: *Rats ingest between 50 and 65% of their feces (82)*.

Incorrect: *Rats ingest between 50 and 65 of their feces (82)*.

In the section “7.6. Serum Levels in Humans”, page 6, left column, 2nd paragraph, 9th line, should read:

Correct: *MS/MS measurements reported that T1AM concentrations in human sera or plasma are far below 1 nM (70). Roy et al. proved excellent stability of deuterated and non-deuterated T1AM in pooled human serum by incubating it for 24 hours at 37°C (57)*.

Incorrect: *MS/MS measurements reported that T1AM concentrations in human sera or plasma are far below 1 nM (57, 70) proved excellent stability of deuterated and non-deuterated T1AM in pooled human serum by incubating it for 24 h at 37°C*.

In the section “8. CONCLUSION”, page 6, right column, the last sentence should read:

Correct: *One can agree with Hoefig et al. (16), that it will take much less time today compared to earlier discoveries, to unravel the mysteries of the novel T_4_ metabolite*.

Incorrect: *One can agree with (16) that it will take much less time today compared to earlier discoveries, to unravel the mysteries of the novel T_4_ metabolite*.

The publisher apologizes for these mistakes. These errors do not change the scientific conclusions of the article in any way.

The original article was updated.

## Conflict of Interest Statement

The authors declare that the research was conducted in the absence of any commercial or financial relationships that could be construed as a potential conflict of interest.

